# The Contextual Effect of Area-Level Unemployment Rate on Lower Back Pain: A Multilevel Analysis of Three Consecutive Surveys of 962,586 Workers in Japan

**DOI:** 10.3390/ijerph16204016

**Published:** 2019-10-20

**Authors:** Takaaki Ikeda, Kemmyo Sugiyama, Jun Aida, Toru Tsuboya, Ken Osaka

**Affiliations:** 1 Department of Health Policy Science, Graduate School of Medical Science, Yamagata University, Yamagata 990-9585, Japan; t.ikeda0110@gmail.com; 2 Department of International and Community Oral Health, Tohoku University Graduate School of Dentistry, Sendai 980-8575, Japan; aidajun@m.tohoku.ac.jp (J.A.); tsubo828@gmail.com (T.T.); osaka@m.tohoku.ac.jp (K.O.)

**Keywords:** unemployment rate, socioeconomic status, education, occupation, Bayesian approach, E-value, spillover effect

## Abstract

This study examined the associations between area-level unemployment rates and lower back pain using large-scale data provided by the Japanese working population. We analyzed data from a nationally representative, repeated, cross-sectional study across three waves from 2010, 2013, and 2016 in 47 Japanese subnational level areas. Workers aged 18–64 years (*n* = 962,586) were eligible to participate in the study. A multilevel logistic model was used to examine the association between the unemployment rate and lower back pain. The self-report of lower back pain was a dependent variable. The prefecture-level unemployment rate was analyzed as an independent variable, adjusted for individual-level covariates (e.g., gender, age, socioeconomic status). After adjusting for all covariates, the main effect of the prefecture-level unemployment rate was statistically significant: the odds ratio (OR) (95% credible interval (CrI)) was 1.01 (1.002, 1.03). Additionally, the OR (95% CrI) for the interaction between gender and the prefecture-level unemployment rate was 1.02 (1.01, 1.03) indicating that women were more affected by area-level employment status than men. In conclusion, a significant association between the unemployment rate and lower back pain was observed in the Japanese working population. Women were more sensitive to the unemployment rate.

## 1. Introduction

Lower back pain is one of the most common symptoms of all ages [1], and a leading cause of disability globally [2]. An increasing number of research studies are being published which have investigated lower back pain, especially in countries with a higher gross domestic product [3]. A recent systematic review estimated that the global lifetime prevalence of lower back pain was 38.9% [1]. This indicates that more than a third of the global population is affected by this symptom. This high prevalence suggests that lower back pain has a great impact not only on individuals and their family members globally, but also entails annual medical costs of more than 1 billion pounds sterling, and undermines work productivity [4,5,6,7].

Macro-level factors such as area-level socioeconomic status (SES) have been linked to adverse health outcomes, such as all-cause mortality [8,9], self-rated health [8], and poor health-related behaviors [10] among all age groups. Therefore, we believe that individuals’ economic situations have an impact upon public health. Specifically, area-level unemployment rates play an important part in the health of working generations, as previous studies have reported that high area-level unemployment rates are a risk factor for mortality [11,12], diabetes [13], depression [14], poor self-rated health [15,16], and poor health-related behaviors [16,17,18] in the working population. A longitudinal study from the United States reported that the state-level unemployment rate was associated with mortality because of limited access to medical care for financial reasons [12]. With this as the backdrop, it could be similarly hypothesized that there is a contextual effect of higher unemployment rates on lower back pain. However, it is unclear whether the area-level unemployment rate is associated with lower back pain in Japan, where universal public healthcare is available. In Japan, the average unemployment rate was 4.8% in 2010, 3.7% in 2013, and 2.8% in 2016, respectively [19]. The rates varied from 1.7% to 7.5% across prefectures.

It is also unclear as to whether the influence of area-level unemployment rate on lower back pain differs between genders, as reports on gender differences in the associations between the unemployment rate and health-related outcomes have been inconsistent: Van Lenthe and colleagues examined data in the Netherlands and reported that women were less likely to be affected by a higher unemployment rate than were men [11]. Another study from Germany reported that living in a higher unemployment neighborhood was associated with the incidence of Type 2 diabetes mellitus in men, but not women [13]. On the other hand, a cohort study from the United States reported that state-level high unemployment rates were associated with limited access to preventive medical services among women, but not among men [18]. Therefore, this study examines (1) associations between area-level employment rates and lower back pain and (2) whether the influence of the area-level unemployment rate on lower back pain differs between genders, using large-scale data from the Japanese working population (Figure S1).

## 2. Materials and Methods

### 2.1. Study Population

**Figure 1:**
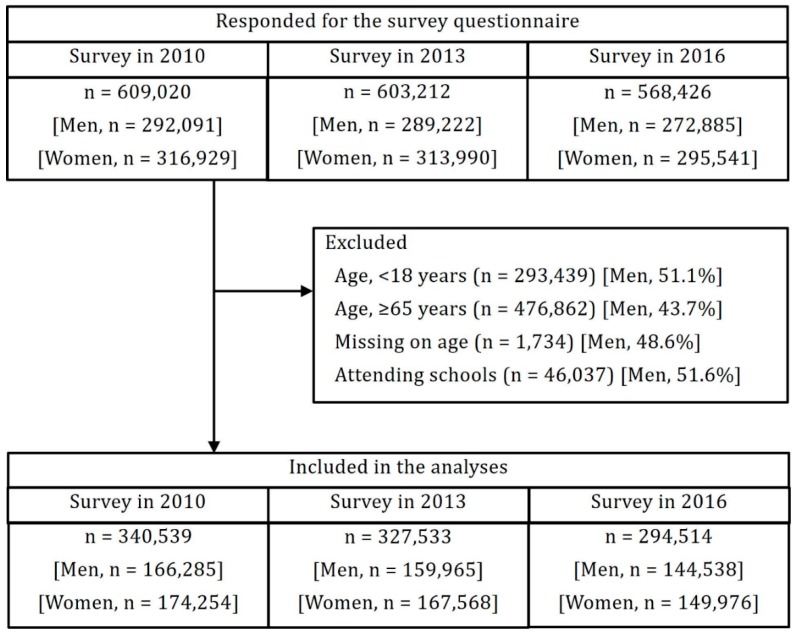
Flow chart diagram of participants for each year of the survey.

We used data from The Comprehensive Survey of Living Conditions of the People on Health and Welfare (CSLC), which is a nationally representative, repeated, cross-sectional, ongoing survey of households and household members conducted by Japan’s Ministry of Health, Labor, and Welfare. The survey was initially conducted in 1986 and has been conducted every three years since to provide information for health assessments at a subnational level in 47 prefectures [20]. Eligible participants were selected through a stratified random sampling method, using districts and the population census [20]. Questions regarding educational attainment were included in the 2010 survey, and we obtained data from three waves: 2010, 2013, and 2016. Surveys covered 289,363 households in 2010 (response rate of 79.4%); 295,367 households in 2013 (response rate of 79.6%); and 289,470 households in 2016 (response rate of 77.6%). In the 2016 survey, Kumamoto Prefecture was excluded from the investigation because of the huge earthquake in 2016. Thus, the number of prefectures examined in 2016 was 46. We also excluded participants (1) who were aged under 18 years (*n* = 293,439) and who were over 65 years (*n* = 476,862); (2) who did not report their age (*n* = 1,734); or (3) who were students (*n* = 46,037). Consequently, data were obtained from 962,586 individuals (340,539 in 2010 (mean age, 44.5 ± 12.6 years); 327,533 in 2013 (mean age, 44.6 ± 12.5 years); and 294,514 in 2016 (mean age, 44.8 ± 12.2 years), were analyzed (Figure 1)).

### 2.2. Statistical Analysis

We used the point prevalence of lower back pain as a dependent variable. Information on lower back pain was obtained from a self-report questionnaire. Participants were asked to choose all symptoms out of the 42 listed if they had experienced them within the past few days. Of these, we identified those who selected lower back pain. We calculated the point prevalence of lower back pain for each wave using a previous systematic review [1].

To measure the contextual effect on lower back pain, we used the prefecture-level unemployment rate as a dependent variable. Information of the prefecture-level unemployment rate was obtained from the Labor Force Survey [19], an ongoing, nationwide survey in Japan, because the CSLC does not have information on the prefecture-level unemployment rate.

We used several covariates on the basis of previous studies [1,21,22,23,24]: age (continuous), gender, educational attainment (≥13 years, <13 years), and occupation (manager, professional, white-collared, blue-collared, and no occupation [including not engaged in an income-earning job]). The cutoff point of educational attainment was also in accordance with previous studies [23,24].

To consider the hierarchical structures in each prefecture and the year in which investigations were conducted, we used multilevel logistic models to evaluate the association of prefecture-level unemployment rates with lower back pain. The data were structured as follows: level 1, individual; level 2, the year of investigation; and level 3, prefectural level [25,26]. Estimates were obtained with Bayesian statistics, using Markov chain Monte Carlo (MCMC) methods. We calculated the estimates with a burn-in of 500 iterations followed by a monitoring chain of 5000 iterations. Due to the different influence of unemployment rates on health-related outcomes between men and women [11,13,18], we also examined models that included the interaction between of gender and unemployment rate.

Before performing the regression analyses, we used multiple imputations of the missing at random assumption for missing values [27]. In our dataset, only variables for educational attainment and occupation included missing values, and the proportion of the missing variables was 8.2%–9.0% and 8.3%–9.2%, respectively. We assumed the pattern of missing variables on the basis of a Little’s test [28]. Missing variables were imputed by multivariate imputation chained equations (MICE) [27] using the following variables: gender, age, educational attainment, occupation, residential prefecture, and year(s) of investigation. Rubin’s rule was used to combine the results across ten imputed datasets [27].

We conducted the same analysis with the complete data, wherein we excluded participants with missing data from the analysis to assess sensitivity. We also conducted sensitivity analyses, wherein we treated all the missing variables on educational attainment and occupation as <13 years and no occupation and as ≥13 years and managers, respectively, and examined the same analysis. In addition, we calculated the E-value to check the robustness of our results to residual confounding factors [29,30]. This value estimates the minimum strength of association between any unmeasured confounder, the area-level unemployment rate, and lower back pain. Using this method, we examined our study effect size besides the odds ratio (OR). In particular, we calculated the E-value for the observed OR using the following formula [29,30]:

E − value = OR + sqrt{OR × (OR − 1)}.

We also performed stratified analyses by gender. We used the Bayesian deviance information criterion (BDIC) to confirm model fit with the complete data.

Multilevel analyses were performed with MLwiN, version 3.02 (Centre for Multilevel Modelling, University of Bristol, Bristol, UK) via Stata, version 15.1 (StataCorp, College Station, TX, USA). All other analyses were conducted using Stata. This study was approved by the ethics committee of Tohoku University (14429).

## 3. Results

**Figure 2:**
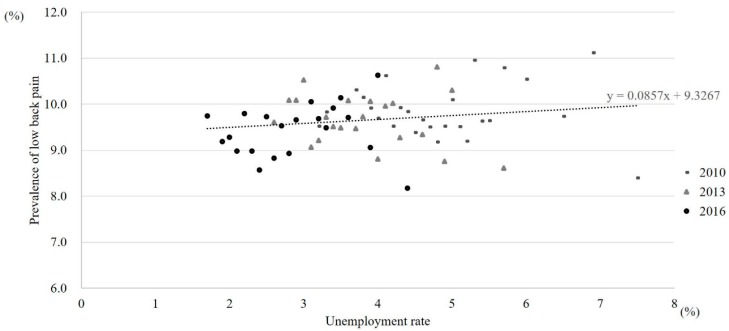
Prevalence of lower back pain and unemployment rate stratified by year of investigation. Each dot represents each prefecture.

**Figure 3:**
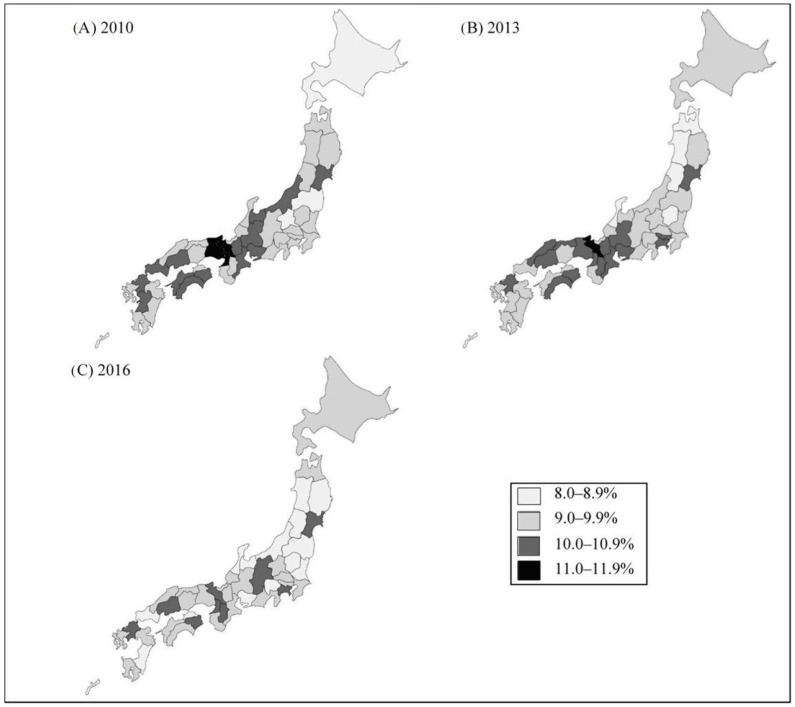
Prevalence of lower back pain and unemployment according to year of investigation and prefecture. (**A**) the 2010 survey; (**B**) the 2013 survey; (**C**) the 2016 survey. In the 2016 survey, data of Kumamoto Prefecture was not collected due to a huge earthquake in 2016.

The prevalence of lower back pain stratified by year of investigation was 9.8% in 2010, 9.7% in 2013, and 9.4% in 2016. Figure 2; Figure 3, as well as Table S1, show the prevalence of lower back pain and the unemployment rate in each prefecture stratified by year of investigation. Overall, both the prefecture-level unemployment rate and the prevalence of lower back pain decreased over time (Figure 2). Besides, the prevalence of lower back pain was relatively higher in the western district of Japan (e.g., Hyogo, Kyoto, Osaka) (Figure 3). Table 1 and Table S2 also show the prevalence of lower back pain that is stratified by each individual-level variable. The prevalence of lower back pain tended to be higher in women, older age groups, those with lower-level educational attainment, and those with lower occupational classification (Table 1). In addition, the number of female managers in our data was substantially lower than that of male managers: 5757 vs. 40,425 (Table S2).

### Unemployment Rate and Prevalence of Lower Back Pain

First, we tested the suitability of random intercepts and random slopes by comparing BDIC in the complete data. Random intercepts provided a better fit than did random slopes and were retained for subsequent analyses. According to the BDIC of the complete data shown in Table S3, Model C provided the best fit in all analyses. Thus, we chose this model’s covariates and interaction terms for our main analysis. Table 2 shows the results of our multilevel analyses of multiply imputed data. After adjusting for all covariates, the main effect of the prefecture-level unemployment rate on lower back pain prevalence was statistically significant: the OR (95% credible interval (CrI)) was 1.01 (1.002, 1.03). In addition, the interaction between gender and the unemployment rate was significant with an OR (95% CrI) of 1.02 (1.01, 1.03). This result means that women were more likely than men to experience lower back pain by 2% for every 1% rise the unemployment rate. Moreover, the following groups were significantly associated with the prevalence of lower back pain: women, older age groups, those with lower-level educational attainment, and those with lower occupational classification (including no occupation).

All results using the complete data were similar to those using data pooled from multiple imputation (Table S3). Overall, being women, being older, having lower educational attainment, and being from a lower occupational class were significantly associated with lower back pain (Table 1 and Table S3). Furthermore, our sensitivity analyses showed similar results with multiple imputation (Table S4). When missing variables for both educational attainment and occupation were treated as <13 years and no occupation, respectively, the association of unemployment rate and lower back pain remained significant: the OR (95% CrI) was 1.02 (1.004, 1.03).

Moreover, the interaction between gender and unemployment rate was significant with an OR (95% CrI) of 1.02 (1.01, 1.03). Similar results were observed when missing variables for both educational attainment and occupation were treated as ≥13 years and manager, respectively: the OR (95% CrI) was 1.01 (1.0003, 1.02), and the interaction between gender and unemployment rate was significant with an OR (95% CrI) of 1.02 (1.01, 1.03).

In addition, the E-value for the point estimate based on our main result (OR [95% CrI] 1.01 [1.002, 1.03]) was 1.11 (lower and upper credible interval limit: 1.05, 1.21). This indicated that the observed OR of 1.01 could be explained by an unmeasured confounder that was associated with both area-level unemployment rate and lower back pain by an OR of 1.11 each, over and above the measured confounder. Tables S5 and S6 also show stratified analyses based on gender. When stratified according to gender, the unemployment rate was statistically associated with lower back pain in both genders; the ORs (95% CrI) were 1.02 (1.004, 1.03) and 1.03 (1.02, 1.04) for men and women, respectively.

## 4. Discussion

We examined whether the prefecture-level unemployment rate was associated with the prevalence of lower back pain over and above the individual factors among the Japanese working population. We found that people living in a prefecture where the unemployment rate was high were more likely to suffer from lower back pain. Moreover, the unemployment rate significantly impacted the female gender’s experience of lower back pain. We also found that each of the conditions of being older; being with lower educational attainment levels; being blue-collared workers; and not having an occupation increased the prevalence of lower back pain.

To the best of our knowledge, our study is the first to reveal an association between the area-level unemployment rate and lower back pain in the working population. A study from England examined the association between area-level deprivation scores, which were calculated using homeownership, car ownership, unemployment, overcrowding, and the onset of chronic widespread pain among the working population [31]. The authors indicated that area-level deprivation scores were positively associated with the incidence of chronic widespread pain [31]; our findings supported this study. We also revealed that the unemployment rate interacted positively with gender. This finding was similar to those of previous studies that indicated gender differences in the effect of area-level SES on health status [11,13,18]. Some might feel that the effect size of the area-level unemployment rate on lower back pain in the present study (OR of 1.01) is small; however, we believe that the magnitude is considerable. Each time the unemployment rate rises by 1%, the number of individuals with lower back pain increases by 770,000 at a moderate estimate [32]. Moreover, the E-value of 1.11 for the contextual effect of area-level SES is moderately large when referring to previous reports included in a systematic review [8]. Thus, we believe that our result is robust when considering the residual contextual confounder.

Moreover, we also revealed that lower levels of educational attainment were significantly associated with the prevalence of lower back pain. Our findings support studies conducted in France [33] and the United States [34]. These two studies reported an association between educational attainment and the prevalence of lower back pain in the general population; our study’s results align with the results of these studies [33,34]. We also found that lower occupational classifications, i.e., blue-collared workers, were significantly associated with the prevalence of lower back pain; this finding was also consistent with earlier studies [35,36]. These findings were consistent with our previous study, which was conducted with the older population [22], and which indicated that the association between individual-level SES and lower back pain was propagative.

One possible pathway could explain the positive association between the area-level unemployment rate and lower back pain. Two studies from the United States reported that the state-level unemployment rate was associated with less preventive care services (e.g., health checkups) [18] and the mortality risk due to limited access to medical care was associated with financial constraints [12]. Another previous study from Italy reported that economic stress mediated the association between the unemployment rate and health-related outcomes, including diabetes and hypertension [16]. Thus, it is possible that the higher unemployment rate limits access to health care services for lower back pain. Moreover, there could be a contextual effect of unemployment rate on lower back pain. The term contextual effect is defined as the influence of environmental factors on a person’s perception of a stimulus, and is one of the important public health topics globally [37]. In particular, a spillover effect is considered to be one possible explanation of the contextual effect, which spreads through physical or social proximity and modifies a person’s behavior [38]. These have both positive and negative effects. In this context, we assume that the area-level unemployment rate has a contextual spillover effect; people living in a higher unemployment rate area are more likely to be affected by the economic situations and health-related behaviors of others living in the same area. That is to say, Japanese people tend to avoid seeing doctors when the unemployment rate rises, due to anxiety and fear of becoming unemployed and to save on the cost of living [12]. This hesitation to consult doctors led to entrenched lower back pain.

Our other finding that women were more likely to be sensitive to the unemployment rate than men was similar to the findings of previous research [18,39]. In addition, we found that Japanese women were more likely to suffer from lower back pain. From a demographic perspective, women’s labor force participation in Japan can be described as an M-shaped curve [40]. This means that the majority leave employment upon marriage or their first birth to commit to childcare. This is much higher in the western countries [41]. Although the tendency of the M-shape curve has improved in Japan [42], women workers after childbirth still are forced to work in an unequal work environment where women are considered responsible for childcare. Japanese women are hired to lower-status positions than men, and the proportion of Japanese women who are not employed is higher than that of Japanese men [40]. In fact, there are more male managers than female, and more women are unemployed than men (Table S2). However, previous studies have indicated that lower back pain was one of the major syndromes affecting women during pregnancy and postpartum [43]. Although female workers are at a higher risk for lower back pain, they would be more likely to stop seeing doctors from financial anxieties if they live in a prefecture where the unemployment rate is relatively high. Thus, we considered that working women in Japan were more likely than men to be affected by the contextual effect of higher area-level unemployment.

Our study has several strengths. First, we analyzed a large, nationally representative dataset (*n* = 962,586) characterized by high response rates (77.6%–79.4%) and covering all the prefectures in Japan. Second, we conducted multilevel analyses using MCMC methods that enabled us to conduct a robust estimation during our examination when compared with the methodology employed in a previous study [31].

However, our study has some limitations that must be considered. First, the presence of lower back pain was determined when participants selected “lower back pain” out of the listed 42 common symptoms in the questionnaire. This self-reported questionnaire with multiple-choice questions might have led to an underestimation of prevalence in the whole study population. However, we reckoned that these underestimations would not differ according to the participants’ characteristics and would have no impact on our present results. Second, information on the severity of pain was lacking in the data that we used. The influence of the unemployment rate on lower back pain might differ according to pain severity. Further studies are required to investigate this possibility. Third, we did not include income, which is another indicator for SES, as a covariate because the survey questionnaire did not provide information about that. However, we included educational attainment and occupation as covariates in our regression models. Fourth, this was a cross-sectional study; therefore, we could not clarify the causal pathway between the unemployment rate and lower back pain, which may mean that lower back pain causes unemployment. However, a previous study reported that area-level SES was associated with the onset of chronic widespread pain [31]. Fifth, the generalizability of our results to other countries and regions remains unclear. Although we conducted a study in Japan only, we considered our data could be generalized to other countries and regions because many previous studies reported associations between higher unemployment rates and poor health-related outcomes [11,12,13,14,15,16,17,18].

## 5. Conclusions

In summary, the present study found a significant association between the unemployment rate and lower back pain among workers in Japan, where universal healthcare coverage is provided. The association was modest; however, it was over and above individual factors. Furthermore, women were more sensitive to the area-level unemployment rate. Policymakers and healthcare workers should consider these findings when tackling inequalities related to lower back pain.

## Figures and Tables

**Table 1:** Individual-level variables and lower back pain.

Individual-Level Variables	2010			2013			2016		
	Participants	Having Lower Back Pain		Participants	Having Lower Back Pain		Participants	Having Lower Back Pain	
	*n*	*n*	%	*n*	*n*	%	*n*	*n*	%
Gender									
Men	166,285	14,784	8.9	159,965	14,003	8.8	144,538	12,334	8.5
Women	174,254	18,685	10.7	167,568	17,719	10.6	149,976	15,289	10.2
Missing	-	-	-	-	-	-	-	-	-
Age									
1st (18–27 years)	39,206	2005	5.1	35,816	1780	5.0	30,380	1403	4.6
2nd (28–33 years)	38,790	2778	7.2	36,015	2562	7.1	30,695	2090	6.8
3rd (34–37 years)	32,783	2593	7.9	29,787	2322	7.8	24,933	1911	7.7
4th (38–41 years)	32,420	2852	8.8	34,835	2993	8.6	29,841	2400	8.0
5th (42–45 years)	30,701	2994	9.8	32,742	2967	9.1	33,277	2952	8.9
6th (46–49 years)	30,232	3150	10.4	29,944	3149	10.5	30,496	2930	9.6
7th (50–54 years)	39,802	4476	11.3	37,710	4329	11.5	35,932	4053	11.3
8th (55–58 years)	35,946	4403	12.3	31,582	3766	11.9	29,463	3527	12.0
9th (59–61 years)	33,026	4291	13.0	26,610	3424	12.9	23,291	2958	12.7
10th (62–64 years)	27,633	3927	14.2	32,492	4430	13.6	26,206	3399	13.0
Missing	-	-	-	-	-	-	-	-	-
Educational attainment									
≥13 years	133,521	11,990	9.0	136,315	11,868	8.7	127,171	11,081	8.7
<13 years	172,778	18,391	10.6	156,659	16,776	10.7	126,835	13,214	10.4
Missing	34,240	3088	9.0	34,559	3078	8.9	40,508	3328	8.2
Occupation									
Manager	16,309	1420	8.7	15,331	1255	8.2	14,542	1170	8.1
Professional	65,342	5991	9.2	65,008	5727	8.8	60,450	5378	8.9
White-collar	100,808	8923	8.9	99,090	8816	8.9	90,779	8006	8.8
Blue-collar	58,861	6471	11.0	54,713	5988	10.9	51,449	5234	10.2
No occupation	74,000	8351	11.3	67,651	7684	11.4	53,375	5839	10.9
Missing	25,219	2313	9.2	25,740	2252	8.8	23,919	1996	8.3

**Table 2:** Association between unemployment rate and lower back pain.

Multiply Imputed Data	Mode1 1			Model 2		
Fixed Effect Parameters	OR	95% CrI		OR	95% CrI	
Individual-level (*n* = 962,586)						
Gender						
Men	1.00			1.00		
Women	1.25	1.23	1.27	1.16	1.11	1.22
Age	1.03	1.02	1.04	1.025	1.024	1.026
Educational attainment						
≥13 years	1.00			1.00		
<13 years	1.09	1.07	1.11	1.09	1.07	1.11
Occupation						
Manager	1.00			1.00		
Professional	1.22	1.17	1.26	1.21	1.17	1.26
White-collar	1.11	1.07	1.15	1.11	1.07	1.15
Blue-collar	1.37	1.32	1.42	1.37	1.32	1.42
No occupation	1.21	1.17	1.26	1.21	1.17	1.26
Prefecture-level (*n* = 47)						
Unemployment rate (continuous)	1.02	1.01	1.04	1.01	1.002	1.03
Interaction term						
Gender × unemployment rate				1.02	1.01	1.03

OR, odds ratio; 95% CrI, 95% credible interval. Model 1, age, gender, educational attainment, and occupation added to Model 1; Model 2, interaction term added to Model 1.

## Supplementary Materials

The following are available online at https://www.mdpi.com/1660-4601/16/20/4016/s1, Figure S1: The hypothesis of our study, Table S1: Prevalence of lower back pain and unemployment rates by year of investigation and prefecture, Table S2: Individual-level variables and lower back pain as a function of gender, Table S3: Associations of individual- and area-level variables with lower back pain in the complete data, Table S4: Sensitivity analyses including occupation type, Table S5: Association between individual- and area-level variables with lower back pain in men in the complete data, Table S6: Association between individual- and area-level variables with lower back pain in women in the complete data.
