# Supplementary material for: The Contextual Effect of Area-Level Unemployment Rate on Lower Back Pain: A Multilevel Analysis of Three Consecutive Surveys of 962,586 Workers in Japan
Source: Int J Environ Res Public Health. 2019 Oct 20;16(20):4016. doi: 10.3390/ijerph16204016 (PMC6843957; doi:10.3390/ijerph16204016)
Supplement: Supplementary file 1 [file ijerph-16-04016-s001.pdf]

International Journal of *Environmental Research and Public Health*

Supplementary Materials

The Contextual Effect of Area-Level Unemployment Rate on Lower Back Pain: A Multilevel Analysis of Three Consecutive Surveys of 962,586 Workers in Japan

Takaaki Ikeda, Kemmyo Sugiyama, Jun Aida, Toru Tsuboya and Ken Osaka

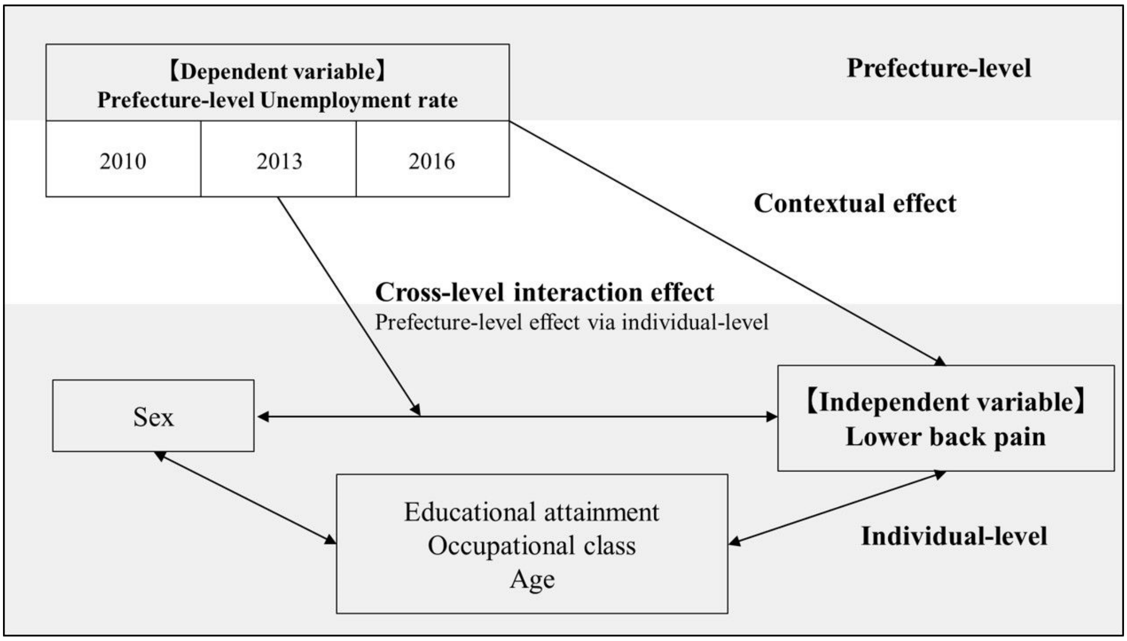

Figure S1. The hypothesis of our study.

Table S1. Prevalence of lower back pain and unemployment rates by year of investigation and prefecture.

	2010		2013		2016	
	**Prevalence (%)**	**Unemployment rate (%)**	**Prevalence (%)**	**Unemployment rate (%)**	**Prevalence (%)**	**Unemployment rate (%)**
Hokkaido	8.6	5.1	9.3	4.6	9.7	3.6
Aomori	9.7	6.5	8.8	4.9	9.1	3.9
Iwate	9.7	5.1	9.4	3.3	8.7	2.4
Miyagi	10.5	5.7	10.5	4.2	10.1	3.2
Akita	9.6	5.4	8.8	4.0	8.3	3.2
Yamagata	9.5	4.5	8.6	3.1	8.0	2.4
Fukushima	8.8	5.2	9.7	3.6	8.3	2.6
Ibaraki	9.0	4.8	9.1	3.9	8.8	2.8
Tochigi	9.8	4.7	8.9	3.7	8.5	2.7
Gunma	8.8	4.7	9.0	3.5	9.2	2.4
Saitama	9.5	5.2	9.9	4.1	9.9	3.2
Chiba	9.3	4.7	9.9	3.7	9.4	2.9
Tokyo	9.6	5.5	10.3	4.2	9.8	3.2
Kanagawa	9.7	4.9	10.1	3.9	10.1	3.1
Niigata	10.2	4.4	9.4	3.5	8.9	2.8
Toyama	10.2	3.8	9.7	2.8	8.9	2.3
Ishikawa	9.8	4.3	8.0	3.2	8.9	2.1
Fukui	9.8	3.3	9.6	2.6	9.2	1.9
Yamanashi	9.3	4.4	9.0	3.1	8.8	2.6
Nagano	9.3	4.0	9.6	3.5	10.5	2.5
Gifu	10.3	3.7	10.5	3.0	9.8	2.2
Shizuoka	9.8	3.9	9.0	3.2	9.3	2.5
Aichi	10.3	4.3	10.1	3.2	8.6	2.4
Mie	10.1	4.0	10.1	2.9	9.2	2.0
Shiga	10.4	4.3	10.5	3.0	9.9	2.5
Kyoto	11.3	5.7	11.0	3.9	10.0	3.1
Osaka	11.1	6.9	10.8	4.8	10.6	4.0
Hyogo	11.0	5.3	10.1	4.1	9.9	3.4
Nara	9.5	4.8	10.4	3.8	10.2	3.2
Wakayama	9.9	4.3	9.8	3.1	9.4	2.0
Tottori	9.5	4.2	10.0	3.4	9.6	2.3
Shimane	9.5	3.2	10.5	2.8	9.7	1.7
Okayama	9.1	4.3	9.7	3.7	9.5	2.7
Hiroshima	10.6	4.1	10.5	3.6	10.2	2.7
Yamaguchi	10.1	3.9	9.1	3.4	8.4	2.4
Tokushima	10.1	4.7	10.2	3.5	10.1	2.7
Kagawa	9.9	3.9	9.7	3.2	8.9	2.6
Ehime	10.0	4.6	9.9	3.4	9.4	2.6
Kochi	10.4	5.0	10.1	3.3	9.5	3.3
Fukuoka	10.5	6.0	10.3	5.0	10.1	3.5
Saga	9.3	4.5	9.0	3.4	9.1	2.1
Nagasaki	9.6	5.0	9.6	4.2	9.9	2.9
Kumamoto	10.3	5.0	9.4	4.2	-	3.1
Oita	9.3	4.6	9.0	3.8	9.7	2.5
Miyazaki	9.0	4.9	9.5	3.7	8.4	2.3
Kagoshima	9.4	5.1	9.3	4.3	9.2	2.8
Okinawa	8.4	7.5	8.6	5.7	8.2	4.4

In the 2016 survey, data of Kumamoto Prefecture was not collected due to a huge earthquake in 2016.

Table S2. Individual-level variables and lower back pain as a function of gender.

	Men			Women		
	Participants	Having lower back pain		Participants	Having lower back pain	
	n	n	%	n	n	%
Age						
1st (18–27 years)	51,901	2,025	3.9	53,501	3,163	5.9
2nd (28–33 years)	51,707	3,211	6.2	53,793	4,219	7.8
3rd (34–37 years)	43,067	3,107	7.2	44,436	3,719	8.4
4th (38–41 years)	47,905	3,799	7.9	49,191	4,446	9.0
5th (42–45 years)	47,208	3,923	8.3	49,512	4,990	10.1
6th (46–49 years)	44,111	3,932	8.9	46,561	5,297	11.4
7th (50–54 years)	55,320	5,395	9.8	58,124	7,463	12.8
8th (55–58 years)	47,307	5,233	11.1	49,684	6,463	13.0
9th (59–61 years)	40,369	4,952	12.3	42,558	5,721	13.4
10th (62–64 years)	41,893	5,544	13.2	44,438	6,212	14.0
Educational attainment						
≥13 years	190,266	14,845	7.8	206,741	20,094	9.7
<13 years	227,224	22,090	9.7	229,048	26,291	11.5
Missing	53,298	4,186	7.9	56,009	5,308	9.5
Occupation						
Manager	40,425	3,324	8.2	5,757	521	9.1
Professionals	112,548	8,783	7.8	78,252	8,313	10.6
White-collar	108,543	8,354	7.7	182,134	17,391	9.6
Blue-collar	121,208	12,388	10.2	43,815	5,305	12.1
No occupation	46,485	4,953	10.7	148,541	16,921	11.4
Missing	41,579	3,319	8.0	33,299	3,242	9.7

Table S3. Associations of individual- and area-level variables with lower back pain in the complete data.

Fixed effect parameters	Model A			Model1 B			Model C		
	OR	95% CrI		OR	95% CrI		OR	95% CrI	
Individual-level (n = 804,122)									
Gender									
Men				1.00			1.00		
Women				1.25	1.23	1.27	1.16	1.10	1.23
Age				1.02	1.02	1.03	1.02	1.02	1.03
Educational attainment									
≥13 years				1.00			1.00		
<13 years				1.10	1.09	1.12	1.10	1.08	1.12
Occupation									
Manager				1.00			1.00		
Professionals				1.22	1.17	1.26	1.22	1.17	1.26
White-collar				1.10	1.06	1.15	1.10	1.06	1.15
Blue-collar				1.40	1.34	1.45	1.39	1.34	1.45
No occupation				1.24	1.20	1.29	1.24	1.20	1.29
Prefecture-level (n = 47)									
Unemployment rate (continuous)	1.02	1.01	1.03	1.02	1.01	1.03	1.01	0.994	1.02
Interaction term									
Gender × unemployment rate							1.02	1.01	1.03
Bayesian DIC	515151.4			506994.8			506987.9		

OR, odds ratio; 95% CrI, 95% credible interval; Bayesian DIC, Bayesian Deviance Information Criterion. Model A, prefecture-level unemployment rate adjusted model; Model B, age, gender, educational attainment, and occupation added to Model A; Model C, interaction term added to Model B.

Table S4. Sensitivity analyses including occupation type.

	Mode1 2-A			Mode1 2-B			Mode1 3-A			Mode1 3-B		
**Fixed effect parameters**	**OR**	**95% CrI**		**OR**	**95% CrI**		**OR**	**95% CrI**		**OR**	95% CrI	
Individual-level (n = 962,586)												
Gender												
Men	1.00			1.00			1.00			1.00		
Women	1.25	1.23	1.27	1.26	1.24	1.28	1.17	1.12	1.23	1.17	1.11	1.23
Age	1.02	1.02	1.03	1.03	1.02	1.03	1.02	1.02	1.03	1.03	1.02	1.03
Educational attainment												
≥13 years	1.00			1.00			1.00			1.00		
<13 years	1.13	1.12	1.15	1.06	1.04	1.07	1.13	1.12	1.15	1.06	1.04	1.07
Occupation												
Manager	1.00			1.00			1.00			1.00		
Professionals	1.16	1.12	1.19	1.23	1.18	1.27	1.16	1.13	1.19	1.23	1.18	1.27
White-collar	1.04	1.02	1.07	1.12	1.08	1.16	1.04	1.02	1.07	1.12	1.08	1.16
Blue-collar	1.30	1.27	1.34	1.42	1.37	1.47	1.31	1.27	1.34	1.42	1.37	1.47
No occupation	1.16	1.13	1.19	1.20	1.16	1.25	1.16	1.13	1.19	1.20	1.16	1.25
Prefecture-level (n = 47)												
Unemployment rate (continuous)	1.02	1.01	1.03	1.03	1.02	1.04	1.01	1.0003	1.02	1.02	1.004	1.03
Interaction term												
Gender × unemployment rate							1.02	1.01	1.03	1.02	1.01	1.03
Bayesian DIC	600412.1			600689.7			600404.9			600683.0		

OR, odds ratio; 95% CrI, 95% credible interval; Bayesian DIC, Bayesian Deviance Information Criterion. Model 2-A and Model 3-A, missing variables on educational attainment and occupation were treated as "≥13 years" and "manager", respectively. Model 2-A added age, gender, educational attainment, occupation, and prefecture-level unemployment rate. Model 3-A added interaction term to Model 2-A. Model 2-B and Model 3-B, missing variables on educational attainment and occupation were treated as "<13 years" and "no occupation", respectively. Model 2-B added age, gender, educational attainment, occupation, and prefecture-level unemployment rate. Model 3-B added interaction term to Model 2-B.

Table S5. Association between individual- and area-level variables with lower back pain in men in the multiply imputed data.

Fixed effect parameters	Model A			Model B		
	**OR**	**95% CrI**		**OR**	95% CrI	
Individual-level (n = 391,971)						
Age				1.03	1.03	1.03
Educational attainment						
≥13 years				1.00		
<13 years				1.10	1.08	1.13
Occupation						
Manager				1.00		
Professionals				1.13	1.09	1.18
White-collar				1.10	1.06	1.16
Blue-collar				1.41	1.35	1.47
No occupation				1.27	1.21	1.33
Prefecture-level (n = 47)						
Unemployment rate (continuous)	1.02	1.004	1.03	1.02	1.004	1.03

OR, odds ratio; 95% CrI, 95% credible interval. Model A, prefecture-level unemployment rate adjusted model; Model B, age, gender, educational attainment, and occupation added to Model A.

Table S6. Association between individual- and area-level variables with lower back pain in women in the multiply imputed data.

Fixed effect parameters	Model A			Model B		
	**OR**	**95% CrI**		**OR**	95% CrI	
Individual-level (n = 412,151)						
Age				1.02	1.02	1.02
Educational attainment						
≥13 years				1.00		
<13 years				1.09	1.07	1.12
Occupation						
Manager				1.00		
Professionals				1.40	1.28	1.53
White-collar				1.17	1.07	1.28
Blue-collar				1.36	1.24	1.49
No occupation				1.27	1.16	1.38
Prefecture-level (n = 47)						
Unemployment rate (continuous)	1.03	1.003	1.04	1.03	1.02	1.04

OR, odds ratio; 95% CrI, 95% credible interval. Model A, prefecture-level unemployment rate adjusted model; Model B, age, gender, educational attainment, and occupation added to Model A.
